# Supplementary material for: The Senotherapeutic Effects of APPA (Apocynin [AP] and Paeonol [PA]) on Senescent Human Chondrocytes
Source: Pharmaceuticals (Basel). 2025 Sep 16;18(9):1386. doi: 10.3390/ph18091386 (PMC12472882; doi:10.3390/ph18091386)
Supplement: Supplementary file 1 [file pharmaceuticals-18-01386-s001.zip › pharmaceuticals-3814235-supplementary.pdf]

Supplementary Materials: The following supporting information can be downloaded at:

Supplementary Figure S1: **APPA did not increase the apoptotic cells in basal condition**

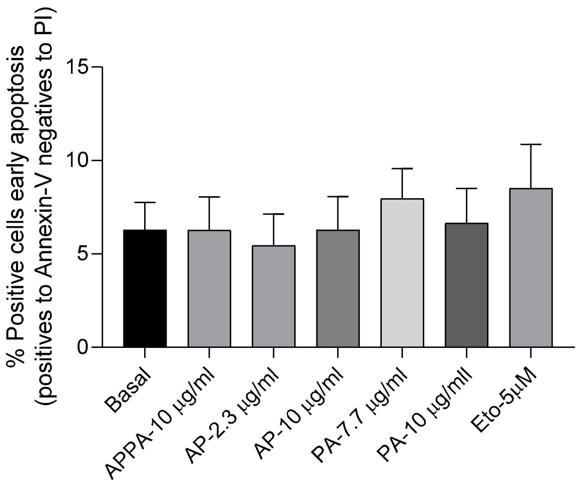

Supplementary Figure S1. APPA effect on Apoptosis. T/C-28a2 human chondrocytes were treated with 5µM Eto + 10 ng/ml OSM for 48 h and with APPA 10 µg/ml, AP (2.3 and 10 µg/ml) and PA (7.7 and 10 µg/ml) during 24 h. Analysis of apoptotic cells was undertaken by flow cytometry; data represented the early apoptosis (cells positive for Annexin-V and negative for PI). All data were obtained from six independent experiments performed with two replicates. Data were represented as mean ± SEM and analyzed by un-paired Mann Whitney test.

Supplementary Figure S2: **APPA effect on Autophagy**

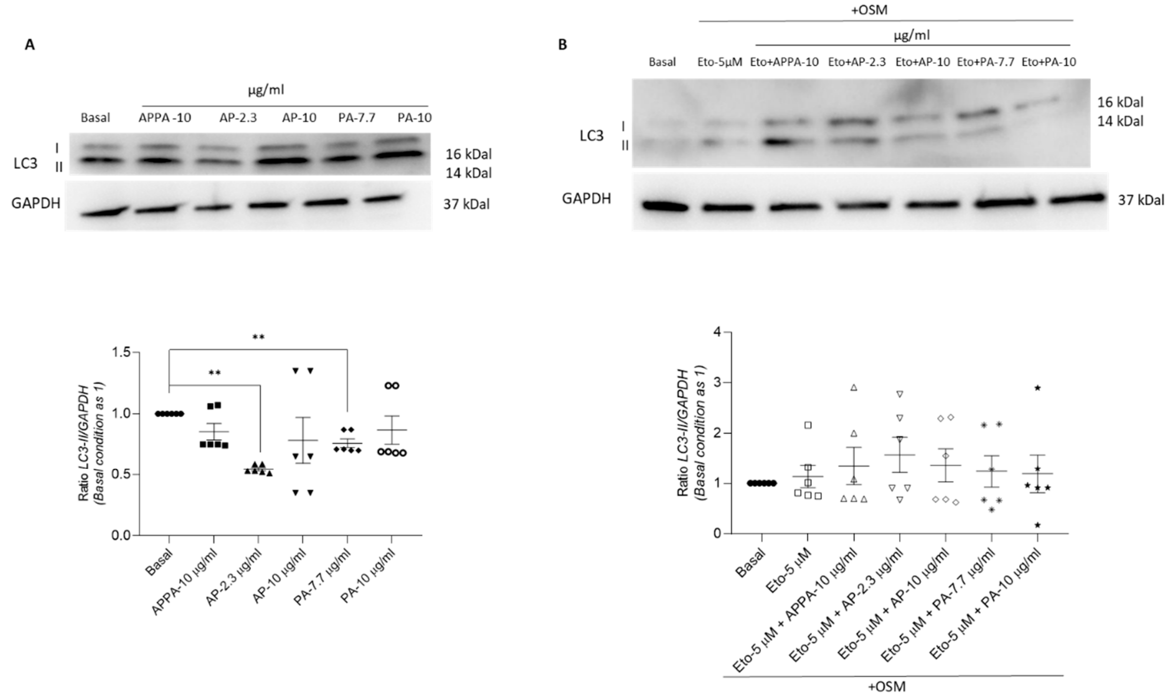

Supplementary Figure S2. APPA effect on Autophagy. Western blotting of protein extracts probed with antibody specific for LC3, and GAPDH Western blotting of protein extracts probed with antibody specific for LC3 and GAPDH. Representative blots were shown along with numeric data obtained by densitometry. **A.** T/C-28a2 human chondrocytes in basal conditions and treated with 10 μg/ml APPA , 2.3 and 10 μg/ml AP and 7.7 and 10 μg/ml PA for 24 h. **B.** T/C-28a2 human chondrocytes treated with Eto 5μM + OSM (10 ng/μl) (Eto) for 48 h and then 10 μg/ml APPA, 2.3 and 10 μg/ml AP and 7.7 and 10 μg/ml PA for 24 h was added. All data were obtained from six independent experiments performed with two replicates. Values are presented as mean ± SEM and analyzed by Mann Whitney test (** $p \leq 0.01$). * relative to basal condition

Supplementary Figure S3: Analysis of cells expressing both senescence and apoptotic markers showed that APPA, AP, and PA had no effect on untreated cells.

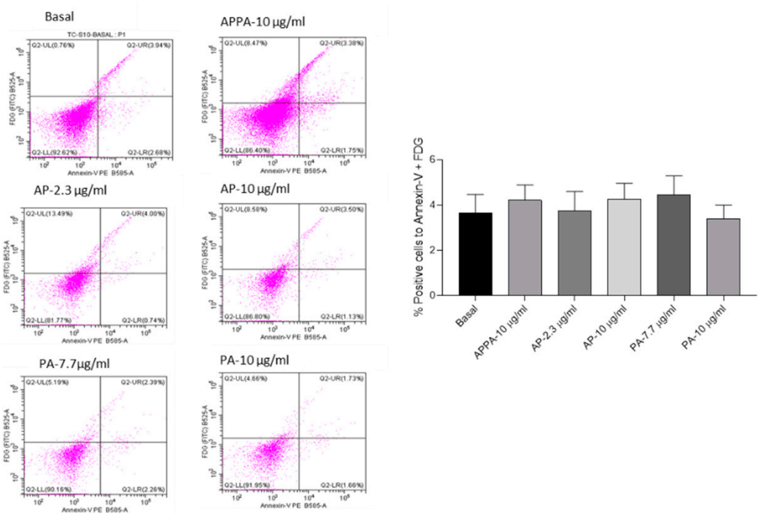

Supplementary Figure S3. Senescence (FDG)/Apoptosis (Annexin-V) double staining was performed on T/C-28a2 cells without treatment (Basal) and treated with the compound evaluated. Representative dot plot for cells in basal condition and treated with APPA 10 µg/ml, AP (2.3 and 10 µg/ml) and PA (7.7 and 10 µg/ml) for 24 h. Graph represented the percentages of FDG/Annexin-V double-positive (upper-right quad-rant_UR_) cells. UL: Senescent cells; UR: Senescent and apoptotic cells; LL: Normal cells (Negative cells); LR: Apoptotic cells. Data are represented as mean ± SEM and analyzed by unpaired Mann Whitney test.

Supplementary Figure S4: APPA, AP and PA on the cells without Eto+OSM treatment´s showed p-rps6 lower modulation.

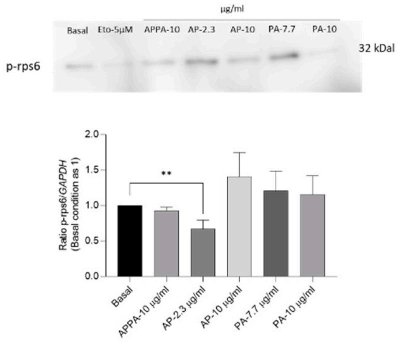

Supplementary Figure S4. Western blotting of protein extracts probed with antibody specific for phospho-ribosomal protein S6 (p-rpS6), and GAPDH. Representative blots were shown along with numeric data obtained by densitometry. Data are represented as mean ± SEM and analyzed by unpaired Mann Whitney test. test (** p≤0.001). * relative to basal condition
